# Bioinformatics identifies key genes and potential drugs for energy metabolism disorders in heart failure with dilated cardiomyopathy

**DOI:** 10.3389/fphar.2024.1367848

**Published:** 2024-03-06

**Authors:** Haixia Wang, Peifeng Cai, Xiaohan Yu, Shiqi Li, Wei Zhu, Yuntao Liu, Dawei Wang

**Affiliations:** ^1^ Guangzhou University of Traditional Chinese Medicine ShunDe Traditional Chinese Medicine Hospital, Guangzhou, China; ^2^ The Second Affiliated Hospital of Guangzhou University of Chinese Medicine, Guangzhou, China; ^3^ Guangdong Provincial Key Laboratory of Clinical Research on Traditional Chinese Medicine Syndrome, China; ^4^ State Key Laboratory of Traditional Chinese Medicine Syndrome/Departments of Gynecologic Oncology, Guangzhou, China; ^5^ The First Affiliated Hospital of Guangzhou University of Chinese Medicine, Guangzhou, China

**Keywords:** bioinformatics, key genes, DCM, heart failure, energy metabolism disorder, NAD^+^, molecular docking, potential drugs

## Abstract

**Background:** Dysfunction in myocardial energy metabolism plays a vital role in the pathological process of Dilated Cardiomyopathy (DCM). However, the precise mechanisms remain unclear. This study aims to investigate the key molecular mechanisms of energy metabolism and potential therapeutic agents in the progression of dilated cardiomyopathy with heart failure.

**Methods:** Gene expression profiles and clinical data for patients with dilated cardiomyopathy complicated by heart failure, as well as healthy controls, were sourced from the Gene Expression Omnibus (GEO) database. Gene sets associated with energy metabolism were downloaded from the Molecular Signatures Database (MSigDB) for subsequent analysis. Weighted Gene Co-expression Network Analysis (WGCNA) and differential expression analysis were employed to identify key modules and genes related to heart failure. Potential biological mechanisms were investigated through Gene Set Enrichment Analysis (GSEA), Gene Ontology (GO), Kyoto Encyclopedia of Genes and Genomes (KEGG), and the construction of a competing endogenous RNA (ceRNA) network. Molecular docking simulations were then conducted to explore the binding affinity and conformation of potential therapeutic drugs with hub genes.

**Results:** Analysis of the left ventricular tissue expression profiles revealed that, compared to healthy controls, patients with dilated cardiomyopathy exhibited 234 differentially expressed genes and 2 genes related to myocardial energy metabolism. Additionally, Benzoylaconine may serve as a potential therapeutic agent for the treatment of dilated cardiomyopathy.

**Conclusion:** The study findings highlight the crucial role of myocardial energy metabolism in the progression of Dilated Cardiomyopathy. Notably, Benzoylaconine emerges as a potential candidate for treating Dilated Cardiomyopathy, potentially exerting its therapeutic effects by targeted modulation of myocardial energy metabolism through NRK and NT5.

## 1 Introduction

Heart Failure (HF) is a chronic and progressive condition that manifests as a clinical syndrome with high morbidity and mortality rates, thereby escalating into a progressively severe public health concern. Dilated Cardiomyopathy (DCM), characterized by the dilation of the left ventricle and compromised systolic function, ranks among the most prevalent causes of HF. It exhibits an estimated prevalence of around 1 in 250–400 individuals in the general population (Seferović et al., 2019). The prevalence is slightly higher in men, with a female to male ratio of approximately 1:1.3 to 1:1.5 (Charron et al., 2018). DCM is mainly characterized by left ventricular dilatation and contractile dysfunction in the absence of hypertensive, valvular, congenital heart disease or significant coronary artery disease (Elliott et al., 2008). The causes of DCM can be categorized into hereditary and non-hereditary, with metabolic disorders being one of the most common pathological underpinnings of non-hereditary DCM (McKenna et al., 2017). Research has highlighted the involvement of diverse mitochondrial proteins engaged in energy metabolism and other functions in the onset and progression of DCM. Mitochondrial dysfunction and disruptions in energy metabolism are believed to contribute to the onset of both DCM and HF (Luczak et al., 2020; Sharma et al., 2020). However, the molecular mechanisms underlying energy metabolism dysfunction in DCM complicated by HF are still not fully understood.

NRK, nicotinamide ribosode kinase, including two subtypes NRK1 and NRK2 (encoded by the Nmrk1 and Nmrk2 genes, respectively) (Bieganowski and Brenner, 2004), a phosphate group transferase that can specifically catalyze the synthesis of nicotinamide riboside (NR) and ATP into nicotinamide mononucleotide (NMN) (He et al., 2022). NMN, as a precursor to nicotinamide adenine dinucleotide (NAD), plays an important role in HF (Zapata-Pérez et al., 2021; Wang et al., 2022). Gene NT5E encodes the ecto-5′-nucleotidase (CD73), which is a glycosyl-phosphatidylinositol (GPI) anchored cell surface enzyme that catalyzes the dephosphorylation of nucleoside 5′-monophosphates, converting it into adenosine (Minor et al., 2019). It imports NR from the external environment into the cell, where NRK converts it into NMN for use in the NAD^+^ salvage pathway (Sociali et al., 2016). Reduced NAD^+^ levels or altered NAD^+^/NADH redox status have been observed in HF (Lee et al., 2016; Yoshino et al., 2018; Hu et al., 2020). Studies suggest that supplementation with NAD^+^ precursors such as NR or NMN may be beneficial for preclinical models or HF patients (Pillai et al., 2010; Diguet et al., 2018; Zhou et al., 2020; Zapata-Pérez et al., 2021; Wang et al., 2022). However, a recent study evaluating published research on human NR supplements found that oral NR supplementation exhibited minimal associated clinical efficacy (Damgaard and Treebak, 2023). This suggests that supplementing with NAD + precursors for the treatment of HF may require further exploration.

Bioinformatics is regularly used in cardiovascular disease research and is anticipated to play a major role in predictive medicine (Khomtchouk et al., 2020; Yu et al., 2023). In this study, a series of bioinformatics analysis techniques were employed to identify hub genes and potential therapeutic drugs related to energy metabolism in DCM with HF, which could provide new insights for the diagnosis and treatment of patients with DCM complicated by HF.

## 2 Materials and methods

### 2.1 Data acquisition and processing

The Gene Expression Omnibus (GEO) database (https://www.ncbi.nlm.nih.gov/geo/) was accessed via the National Center for Biotechnology Information to search for the term “Heart Failure”. Three datasets containing left ventricular samples from patients with DCM complicated by HF and healthy controls were downloaded (Supplementary Table S1). Specifically:

A.The GSE116250 dataset, based on the GPL16791, comprised 14 control samples and 37 DCM samples.

B.The GSE57345 dataset, available on both the GPL9052 and GPL11532, included 139 control samples and 82 DCM samples.

C.The GSE29819 dataset, based on the GPL570, included 6 control samples and 7 DCM samples.

### 2.2 Gene set enrichment analysis (GSEA)

GSEA was conducted using the ‘clusterProfiler’ R package (Yu et al., 2012), with visualization facilitated by the ‘ggplot2’ and ‘enrichplot’ packages. The gene expression values of the samples were analyzed based on the h. all.v7.4. entrez.gmt [Hallmarks] gene set database. Gene sets meeting the criteria of false discovery rat (FDR) < 0.25, Nominal (NOM) *p*-value <0.05, and |Normalized Enrichment Score (NES)| > 1 were considered significantly enriched.

### 2.3 Identification of differentially expressed genes (DEGs)

Identification of DEGs: Differential expression analysis was performed using the ‘limma’ R package. Genes with FDR <0.25 and |logFC| > 0.585 were classified as DEGs. The ‘pheatmap’ and ‘ggplot2’ packages were used to create heatmaps and volcano plots of DEGs for visualization, respectively.

### 2.4 Gene ontology (GO) and kyoto encyclopedia of genes and genomes (KEGG) functional enrichment analysis

The ‘clusterProfiler’ R package was utilized for GO and KEGG functional enrichment analyses. These analyses were performed to assess gene-related biological processes (BP), molecular functions (MF), cellular components (CC), and gene-related signaling pathways.

### 2.5 The weighted gene co-expression network analysis (WGCNA)

To investigate the co-expression relationships among genes and their associations with phenotypes, the ‘WGCNA’ R package was utilized for construction (Langfelder and Horvath, 2008). After outlier samples were removed based on the clustering tree, the top 5,000 genes with a median absolute deviation (MAD) > 1 were selected. A correlation matrix between genes was computed to establish a similarity matrix. To ensure a scale-free network construction, an appropriate soft threshold was chosen to transform the similarity matrix into an adjacency matrix. A topological overlap matrix (TOM) was then created to measure the average network connectivity of each gene. The dynamic tree cutting process was used to cluster genes with similar expression profiles into distinct modules, involving parameter settings such as minModuleSize and mergeCutHeight within the blockwiseModules function. Each resulting module was visually distinguished by a unique color, while genes in the gray module were not assigned to any other modules.

The gene expression profile of each module was captured by its first principal component, referred to as the module eigengene (ME). Using MEs, the correlation between modules and phenotypes was assessed. The module with the highest absolute correlation coefficient was identified as the key module for further analysis. Module membership (MM) represents the correlation coefficient between a gene’s expression value and the ME of the module, providing insight into the correlation between the gene and the module. On the other hand, Gene significance (GS) indicates the correlation coefficient between a gene’s expression value and the expression level of the dependent variable, reflecting the correlation between the gene and the phenotype.

### 2.6 Energy metabolism-related genes

Twenty-three energy metabolism-related gene sets were extracted from the Molecular Signatures Database (MSigDB) v7.5.1, including GOBP_ENERGY_DERIVATION_BY_OXIDATION_OF_ORGANIC_COMPOUNDS, GOBP_ENERGY_RESERVE_METABOLIC_PROCESS, GOBP_ATP_SYNTHESIS_COUPLED_ELECTRON_TRANSPORT,GOBP_GENERATION_OF_PRECURSOR_METABOLITES_AND_ENERGY, GOBP_AEROBIC_RESPIRATION, GOBP_GLUCOSE_METABOLIC_PROCESS, GOBP_NEGATIVE_REGULATION_OF_OXIDATIVE_PHOSPHORYLATION, GOBP_OXIDATIVE_PHOSPHORYLATION, GOBP_REGULATION_OF_GENERATION_OF_PRECURSOR_METABOLITES_AND_ENERGY, GOCC_PROTON_TRANSPORTING_TWO_SECTOR_ATPASE_COMPLEX, KEGG_FATTY_ACID_METABOLISM, MOOTHA_MITOCHONDRIA, KEGG_PYRUVATE_METABOLISM, MOOTHA_VOXPHOS, REACTOME_FATTY_ACID_METABOLISM, REACTOME_GLUCOSE_METABOLISM, REACTOME_INTEGRATION_OF_ENERGY_METABOLISM, REACTOME_PYRUVATE_METABOLISM, REACTOME_PYRUVATE_METABOLISM_AND_CITRIC_ACID_TCA_CYCLE, WP_ENERGY_METABOLISM, WP_NAD_METABOLISM, BIOCARTA_ETC.,_PATHWAY. After removing the overlapping genes, the gene sets associated with energy metabolism contained 645 genes.

### 2.7 Receiver operating characteristic (ROC) curve analysis

The ‘pROC’ R package was utilized to generate ROC curves (Robin et al., 2011), assessing the diagnostic value of key genes associated with HF energy metabolism. Genes that achieved AUC scores in the range of 0.7–1.0 were considered to exhibit excellent specificity and sensitivity.

### 2.8 LncRNA/circRNA-miRNA-NRK and LncRNA/circRNA-miRNA-NT5E regulatory network analysis

Databases such as ENCORI, miRabel, miRDB, miRwalk, and TargetScan were employed to predict interactions between non-coding RNAs and mRNAs. In particular, the ENCORI database was used to predict interactions involving long non-coding RNAs (lncRNAs) and circular RNAs (cricRNAs) with microRNAs (miRNAs). To visualize and illustrate the interactions within the competitive endogenous RNA (ceRNA) network, encompassing lncRNA/cricRNA-miRNA-mRNA interactions, the Cytoscape software (version 3.8.2) was utilized for network construction and visualization.

### 2.9 Single-cell analysis

To investigate the distribution of hub genes across cell groups, a database was utilized for the analysis and visualization of results. The initial source of single-cell RNA sequencing data from DCM with HF was the GSE183852 dataset.

### 2.10 Molecular docking

The three-dimensional crystal structures of the two targets were obtained from the Research Collaboratory for Structural Bioinformatics (RCSB) Protein Database (PDB) (Berman et al., 2000). Similarly, the three-dimensional structures of the two compounds were retrieved from the PubChem database. Hydrogen atoms were added and charges were calculated for both small molecular ligands and target proteins using AutoDock Tools 1.5.7 software (Morris et al., 2009). Molecular docking was then performed via AutoDock Vina 1.1.2 (Trott and Olson, 2010), with the aim of identifying complexes formed between small molecular ligands and protein receptors, prioritizing configurations with the lowest binding energy. The results of the molecular docking procedure were subsequently visualized using PyMOL 2.5.4 software.

### 2.11 Statistical analysis

All data processing and analytical procedures were conducted using the R programming language (version 4.3.0). In the analysis of mRNA and microRNA expression levels, the unpaired Student’s T-test was utilized for data conforming to a normal distribution, while the Wilcoxon rank-sum test was applied to data that did not follow a normal distribution, and *p* < 0.05 was considered statistically signifcant. All other statistical analysis methods were conducted using corresponding R packages, which have been introduced in the Materials and Methods.

## 3 Results

### 3.1 Data preprocessing and DEGs screening

**FIGURE 1:**
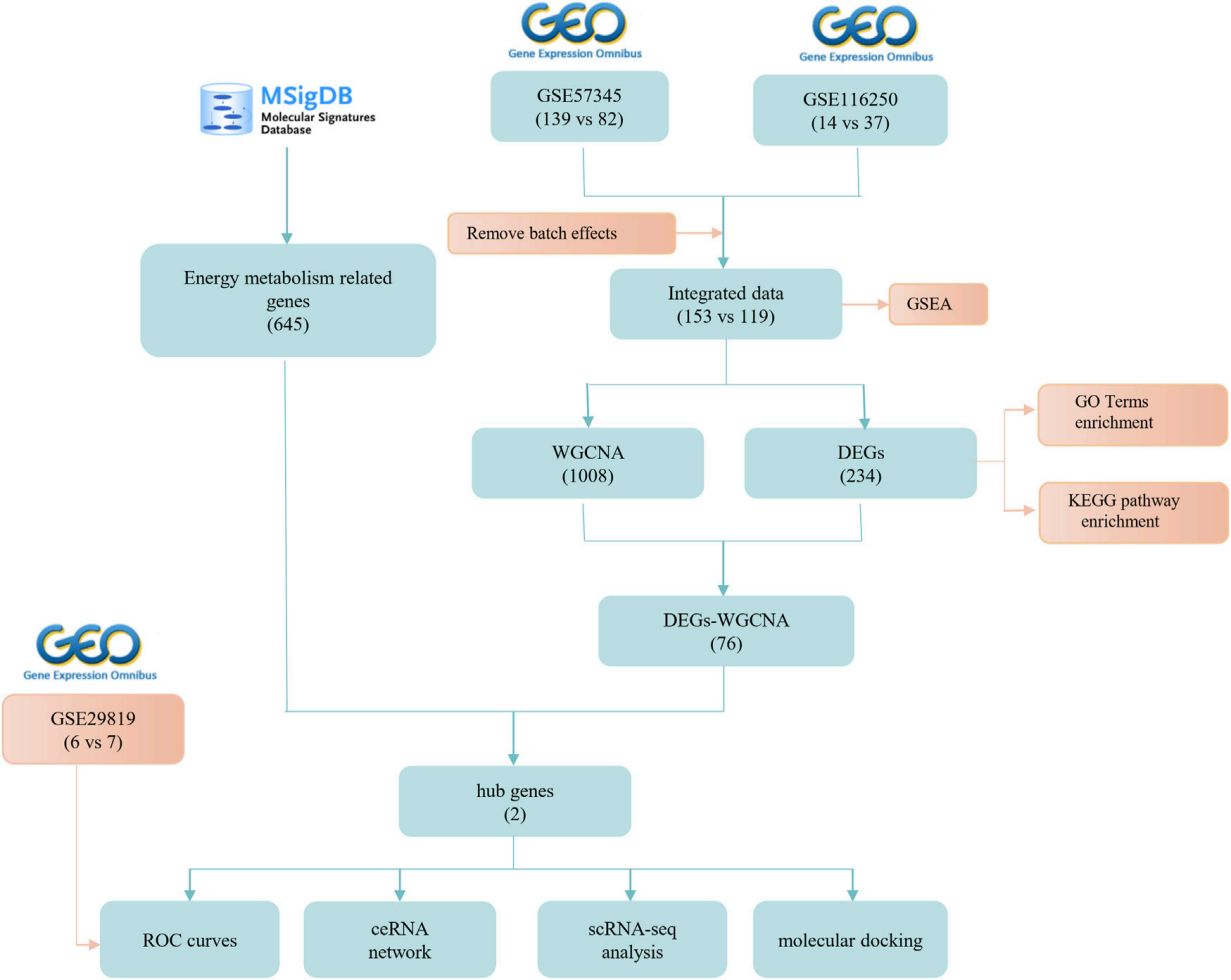
Flow chart of the research process.

**FIGURE 2:**
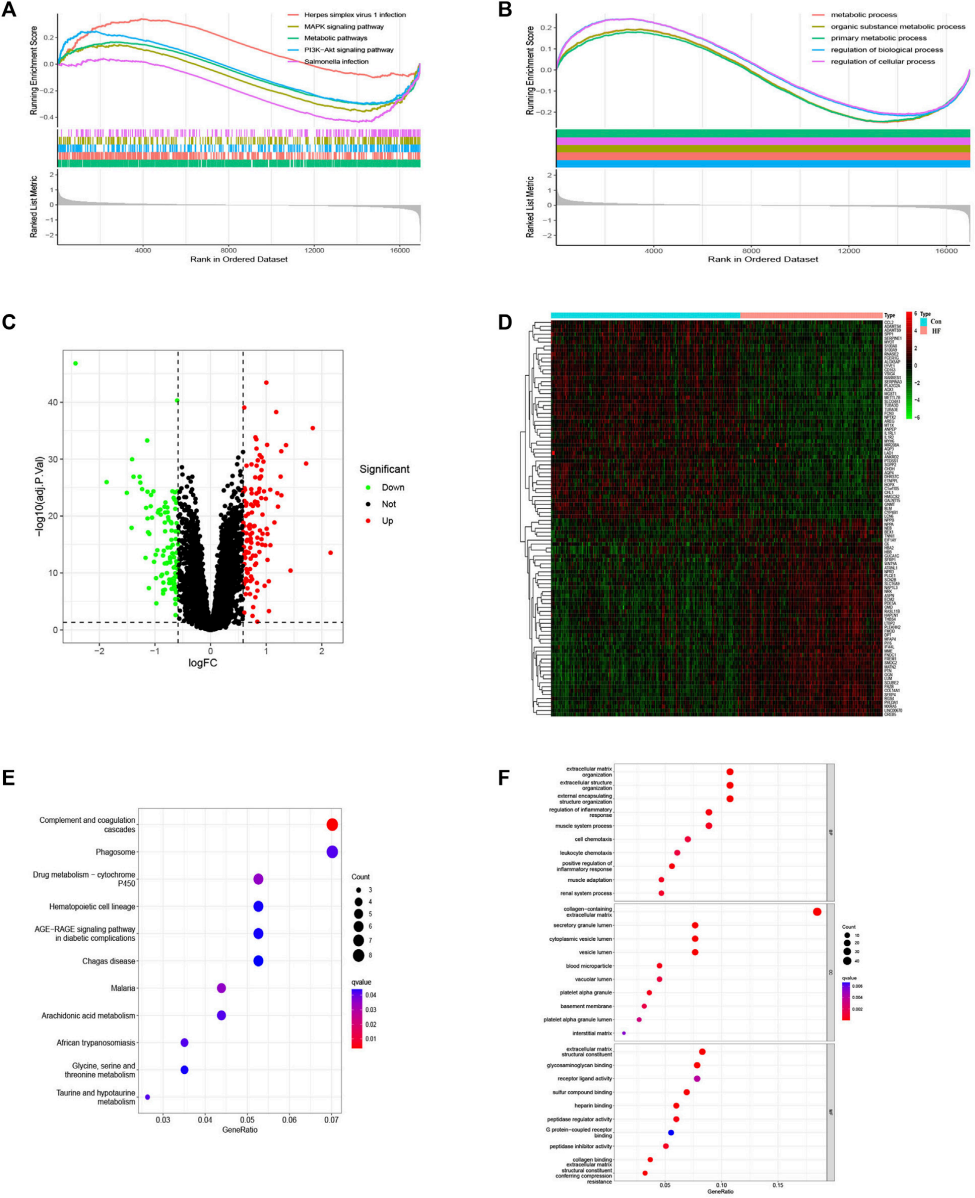
GSEA of the top 5 enriched pathways, biological process and analysis of DEGs. **(A)** KEGG pathway enrichment ananlysis. **(B)** BP enrichment analysis. **(C)** Volcanic map of DEGs. **(D)** Heat map of DEGs. **(E)** KEGG term analysis of DEGs. **(F)** GO term analysis of DEGs.

The bioinformatics workflow of this study is depicted in Figure 1. Batch effects were eliminated from the GEO dataset to yield an integrated dataset (Supplementary Figure S1), comprising 119 samples of DCM with HF and 153 control samples. To identify changes in gene expression associated with HF accompanying DCM, we conducted a differential gene expression analysis. Application of GSEA to normalized raw data revealed that metabolic pathways and processes were notably enriched among the pathways and biological processes (Figures 2A, B). A subsequent differential analysis on gene expression between HF samples and control samples unveiled 234 DEGs, with 128 being upregulated and 106 downregulated (Figures 2C, D). These DEGs were then subjected to GO and KEGG pathway enrichment analysis using the clusterProfiler packages in the R software environment. The KEGG pathway analysis (Figure 2E) revealed significant enrichment in pathways like glycine, serine, and threonine metabolism, as well as involvement in the phagosome pathway. The enriched GO terms analysis (Figure 2F) highlighted the association of these genes with terms such as extracellular matrix organization (GO:0030198), collagen-containing extracellular matrix (GO:0062023), and extracellular matrix structural constituent (GO:0005201). The findings suggest that there may be alterations in cellular metabolism in patients with HF accompanied by DCM compared to healthy individuals.

### 3.2 Constructing a WGCNA to identify key modules associated with HF

**FIGURE 3:**
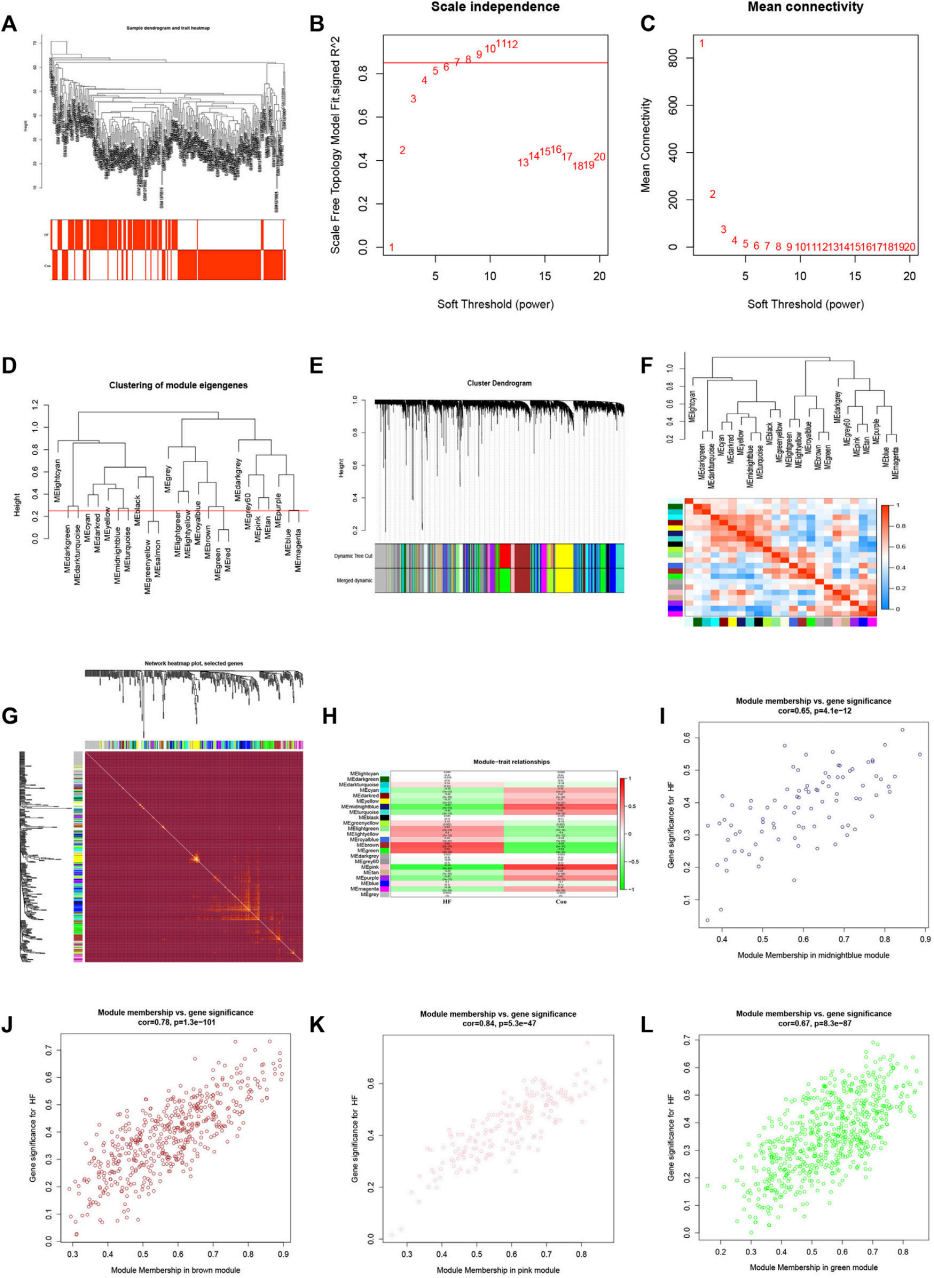
WGCNA consruction and identification of key modules. **(A)** Sample clustering dendrogram with tree leaves corresponding to individual samples. **(B, C)** Soft threshold β = 7 and s. cale-free topological fit index (*R* ^2^). **(D)** Clustered dendrograms were cut at a height of 0.25 to detect and combine similar modules. **(E)** Shows the original and combined modules under the clustering tree. **(F)** Collinear heat map of module feature genes. Red color indicates a high correlation, blue color indicates opposite results. **(G)** Clustering dendrogram of module feature genes. **(H)** Heat map of module-trait correlations. **(I)** The scatterplot describing the relationship between MM and GS in midnightblue module. **(J)** The scatterplot describing the relationship between MM and GS in brown module. **(K)** The scatterplot describing the relationship between MM and GS in pink module. **(L)** The scatterplot describing the relationship between MM and GS in green module.M.

To identify the gene clusters most closely associated with HF, we conducted a WGCNA. The application of WGCNA led to the identification of co-expression modules containing genes that exhibited both robust co-expression levels and high topological overlap similarity. Sample clustering was conducted using Pearson’s correlation coefficient, resulting in the generation of a sample clustering tree (Figure 3A). To construct a network adhering to scale-free topology principles, a soft-threshold of 7 was chosen, guided by an R-squared (R2) value of 0.85. This was followed by the transformation of the adjacency matrix into a Topological Overlap matrix (Figures 3B, C), characterizing node similarity while incorporating weighted correlations.

By setting a clustering height limit of 0.25, 23 modules were identified after merging closely associated modules (Figure 3D). These primed and merged modules were visually represented under the clustering tree (Figure 3E). An assessment of the correlation between modules revealed no significant association between them (Figure 3F). The reliability of module delineation was confirmed by transcription correlation analysis within modules, which showed no substantial linkage between modules (Figure 3G). Further investigation using frontal correlations between ME values and clinical features unveiled intriguing associations. The pink module displayed a positive correlation with the control group (r = 0.74, *p* = 2e−47) and a negative association with HF (r = −0.74, *p* = 2e−47). Conversely, the brown module showed a negative correlation with the control group (r = −0.68, *p* = 5e−37) and a positive association with HF (r = 0.68, *p* = 5e−37). The green module exhibited a negative correlation with the control group (r = −0.64, *p* = 3e−32) and a positive association with HF (r = 0.64, *p* = 3e−32). Lastly, the midnightblue module demonstrated a positive connection with the control group (r = 0.63, *p* = 1e−31) and a negative correlation with HF (r = −0.63, *p* = 1e−31) (Figure 3H). These clinically meaningful modules, namely, pink, brown, green, and midnightblue, were found to be highly associated with HF in the MM *versus* GS scatter plot (Figures 3I–L). Each module contained 126, 453, 335, and 94 key genes, respectively. This suggests that the occurrence of HF is highly correlated with these 1,008 genes.

### 3.3 Identification of hub gene expression levels and diagnostic value

**FIGURE 4:**
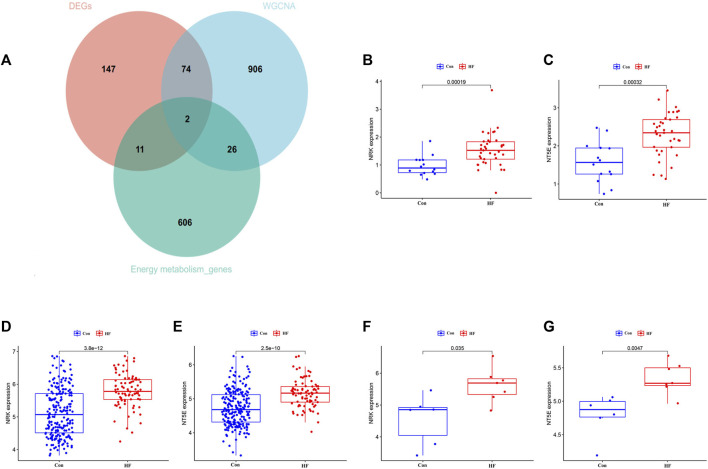
The expression leve of hub genes. **(A)** Two hub genes were screened. **(B)** Expression level of NRK in GSE116250 dataset. **(C)** Expression level of NT5E in GSE116250 dataset. **(D)** Expression level of NRK in GSE57345 dataset. **(E)** Expression level of NT5E in GSE57345 dataset. **(F)** Expression level of NRK in GSE29819 dataset. **(G)** Expression level of NT5E in GSE29819 dataset.

To identify key genes and determine their diagnostic value, we conducted the following analyses. The intersection of DEGs associated with energy metabolism and genes from the four modules correlated with HF led to the identification of two hub genes, namely, NRK and NT5E (Figure 4A). The expression levels of these two hub genes were identified through box plots. As depicted in Figures 4B–E, both NRK (*p* = 0.00019, *p* = 2.5e−10) and NT5E (*p* = 0.00012, *p* = 3.8e−12) demonstrated significantly elevated expression levels in HF compared to the control group. To corroborate the findings in the training datasets, the expression levels of NRK and NT5E were examined in the GSE29819 dataset (Figures 4F, G).

**FIGURE 5:**
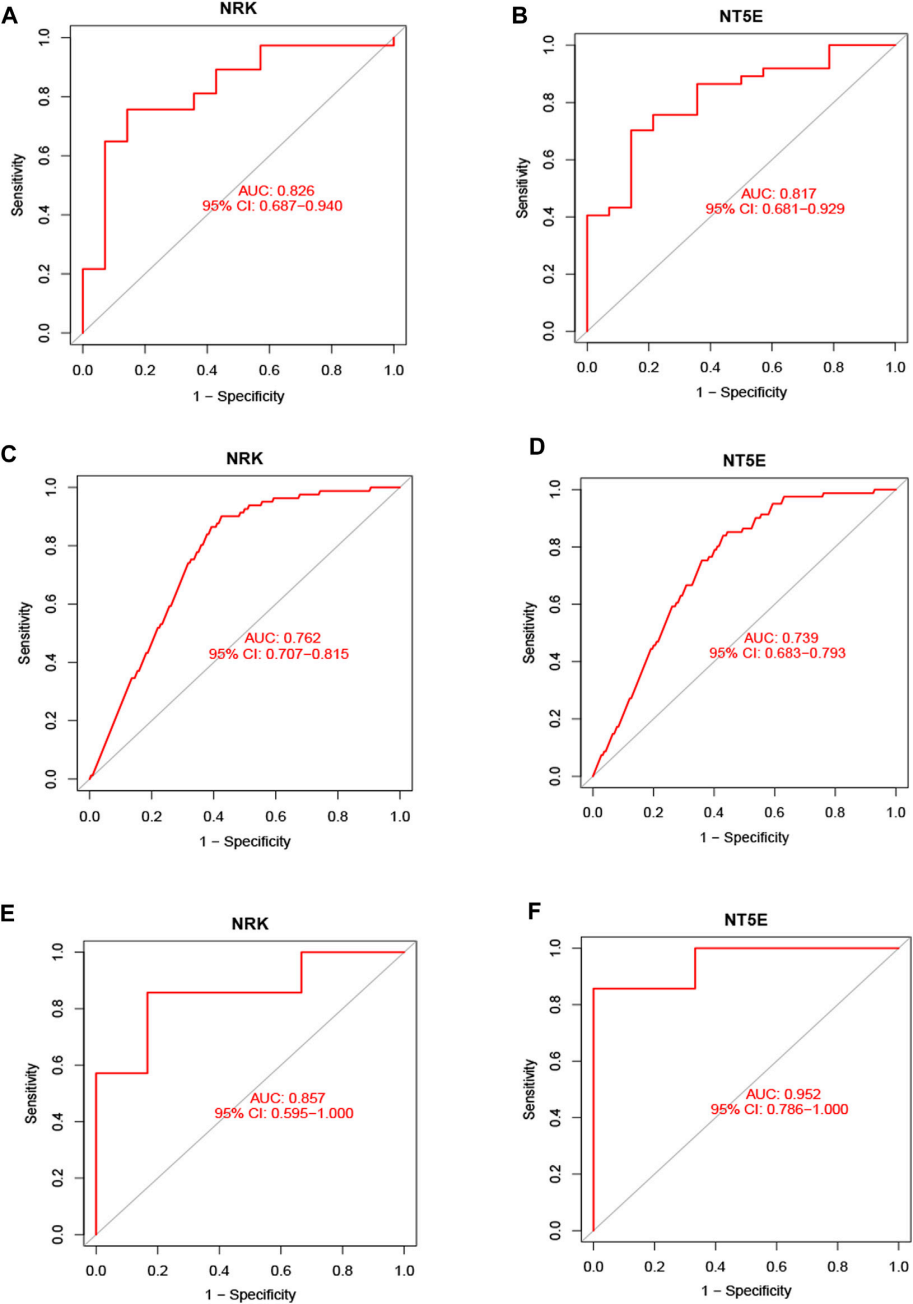
Validation of hub genes in the diagnostic value. **(A)** Validation the hub gene NRK in GSE116250. **(B)** Validation the hub gene NT5E in GSE116250. **(C)** Validation the hub gene NRK in GSE57345. **(D)** Validation the hub gene NRK in GSE57345. **(E)** Validation the hub gene NRK in GSE29819. **(F)** Validation the hub gene NT5E in GSE29819.

Subsequently, ROC curve analysis was conducted to evaluate the diagnostic value of the two hub genes in identifying energy metabolism disorders in DCM with HF. The AUC values were computed to assess their sensitivity and specificity. In the GSE116250 dataset, the AUC of ROC for NRK and NT5E were 0.826 and 0.817, respectively (Figures 5A, B). In the GSE57345 dataset, the AUC of ROC for NRK and NT5E were 0.762 and 0.739, respectively (Figures 5C, D). The diagnostic value of the two hub genes was further validated in the GSE29819 dataset. The AUC values of ROC were 0.857 for NRK and 0.952 for NT5E (Figures 5E, F). These results suggest that the identified hub genes possess significant diagnostic efficiency in predicting energy metabolism disorders in HF with DCM.

### 3.4 CeRNA analysis

In the realm of energy metabolism disorders associated with DCM and HF, recent research has underscored the role of lncRNAs in modulating downstream mRNA expression by targeting miRNAs (Zhou et al., 2019; Larocca et al., 2020). This study aimed to explore the ceRNA network that may regulate the expression of NRK and NT5E in HF with DCM.

**FIGURE 6:**
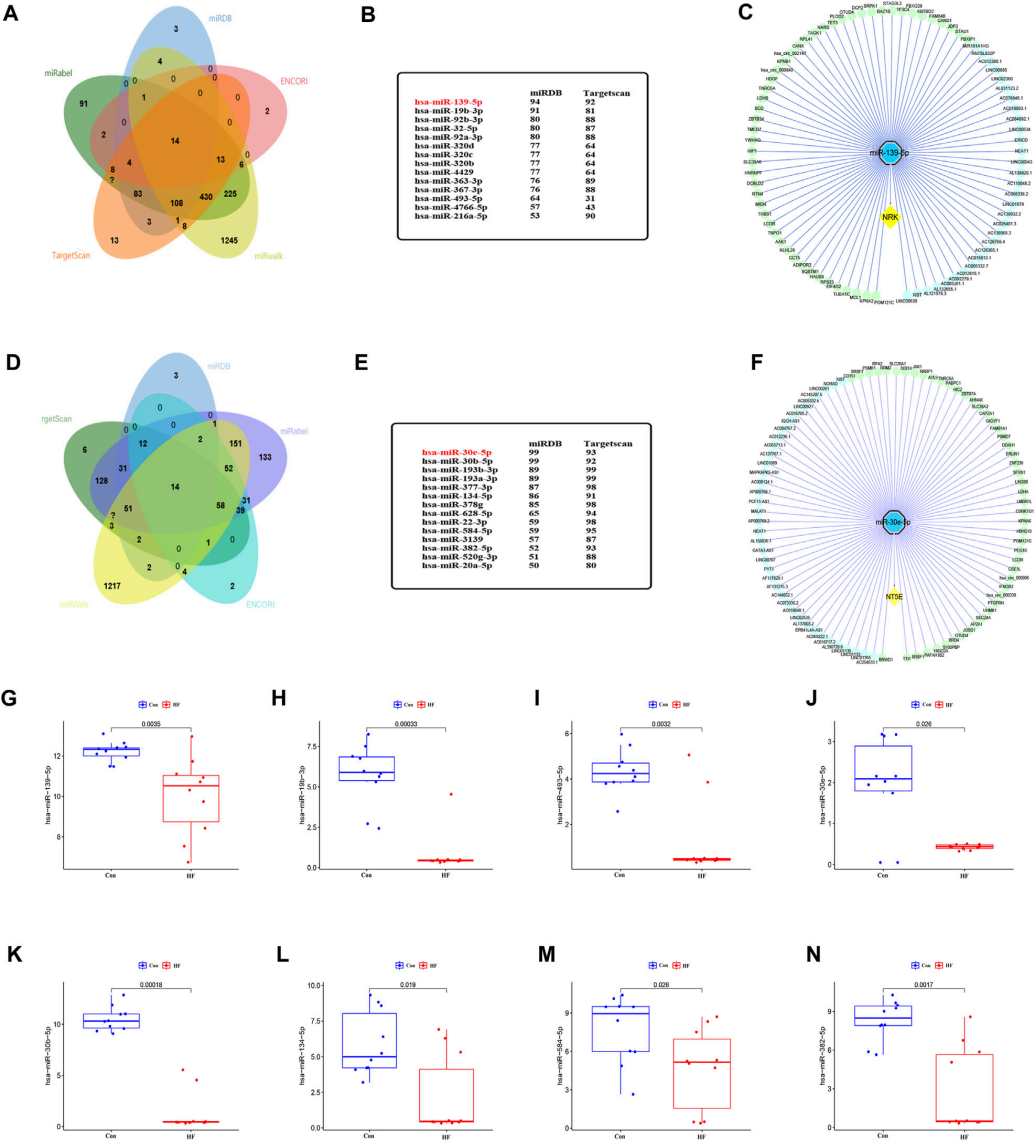
The lncRNA/circRNA-miRNA-NRK and lncRNA/circRNA-miRNA-NT5E regulatory networks constructed. **(A)** The upstream miRNAs of NRK were predicted by ENCORI, miRWalk, miRDB, Targetscan and miRabel databases and the intersection was taken (14 intersection miRNAs). **(B)** The miRNAs targeting NRK were displayed in miRDB and Targetscan database. **(C)** The lncRNA/circRNA-hsa-miR-139-5p-NRK regulatory network was constructed by Cytoscape. circRNA, green nodes; lncRNA, wathetblue nodes; miRNA, blue nodes; mRNA, yellow nodes. **(D)** The upstream miRNAs of NT5E were predicted by ENCORI, miRWalk, miRDB, Targetscan and miRabel databases and the intersection was taken (14 intersection miRNAs). **(E)** The miRNAs targeting NT5E were displayed in miRDB and Targetscan database. **(F)** The lncRNA/circRNA-hsa-miR-30e-5p-NT5E regulatory network was constructed by Cytoscape. circRNA, green nodes; lncRNA, wathetblue nodes; miRNA, blue nodes; mRNA, yellow nodes. **(G)** Expression level of hsa-miR-139-5p. **(H)** Expression level of hsa-miR-19b-3p. **(I)** Expression level of hsa-miR-493-5p. **(J)** Expression level of hsa-miR-30e-5p. **(K)** Expression level of hsa-miR-30b-5p. **(L)** Expression level of hsa-miR-134-5p. **(M)** Expression level of hsa-miR-584-5p. **(N)** Expression level of hsa-miR-382-5p.

To identify miRNAs that may target NRK and NT5E mRNA, we conducted a comprehensive screening of several databases, including miRWalk, miRDB, ENCORI, Targetscan, and miRabel. This led to the identification of 14 miRNAs with potential to target NRK mRNA and another 14 that could potentially target NT5E mRNA (Figures 6A, D). The group of NRK-targeting miRNAs included hsa-miR-139-5p, hsa-miR-19b-3p, hsa-miR-92b-3p, hsa-miR-32-5p, among others (Figure 6B). On the other hand, the NT5E-targeting miRNAs comprised of hsa-miR-30e-5p, hsa-miR-30b-5p, hsa-miR-193b-3p, hsa-miR-193a-3p, and others (Figure 6E). To validate the accuracy of these predictions, we utilized the GSE209991 dataset from the GEO database. This dataset includes miRNA data from 10 donors and 10 DCM patients. A differential analysis revealed that several predicted miRNAs such as hsa-miR-139-5p, hsa-miR-19b-3p, hsa-miR-493-5p, hsa-miR-30e-5p, hsa-miR-30b-5p, hsa-miR-134-5p, hsa-miR-584-5p, and hsa-miR-382-5p were downregulated in the plasma of DCM patients (Figures 6G–N).

Subsequently, we performed predictions in the ENCORI database for upstream lncRNAs and circRNAs that could potentially interact with hsa-miR-139-5p and hsa-miR-30e-5p. We discovered 31 lncRNAs and 50 circRNAs that could competitively bind to NRK with hsa-miR-139-5p. Additionally, we found 39 lncRNAs and 50 circRNAs that could competitively bind to NT5E with hsa-miR-30e-5p (Figures 6C, F). These findings suggest the existence of an upstream ceRNA regulatory network that may contribute to the dysregulated expression of NRK and NT5E in DCM with HF.

### 3.5 Expression of NRK and NT5E in HF with DCM

In an effort to understand the mechanisms underlying DCM leading to HF, especially those involving the structural and energy metabolism aspects of myocardial cells, we concentrated on the expression levels of hub genes in cardiomyocytes (Vignoli et al., 2022). We conducted a single-cell RNA-seq analysis using the GSE183852 dataset to examine the expression patterns of NRK and NT5E in DCM patients with HF. Quality control, data cleaning, and principal component analysis were carried out as shown in Supplementary Figure S2.

**FIGURE 7:**
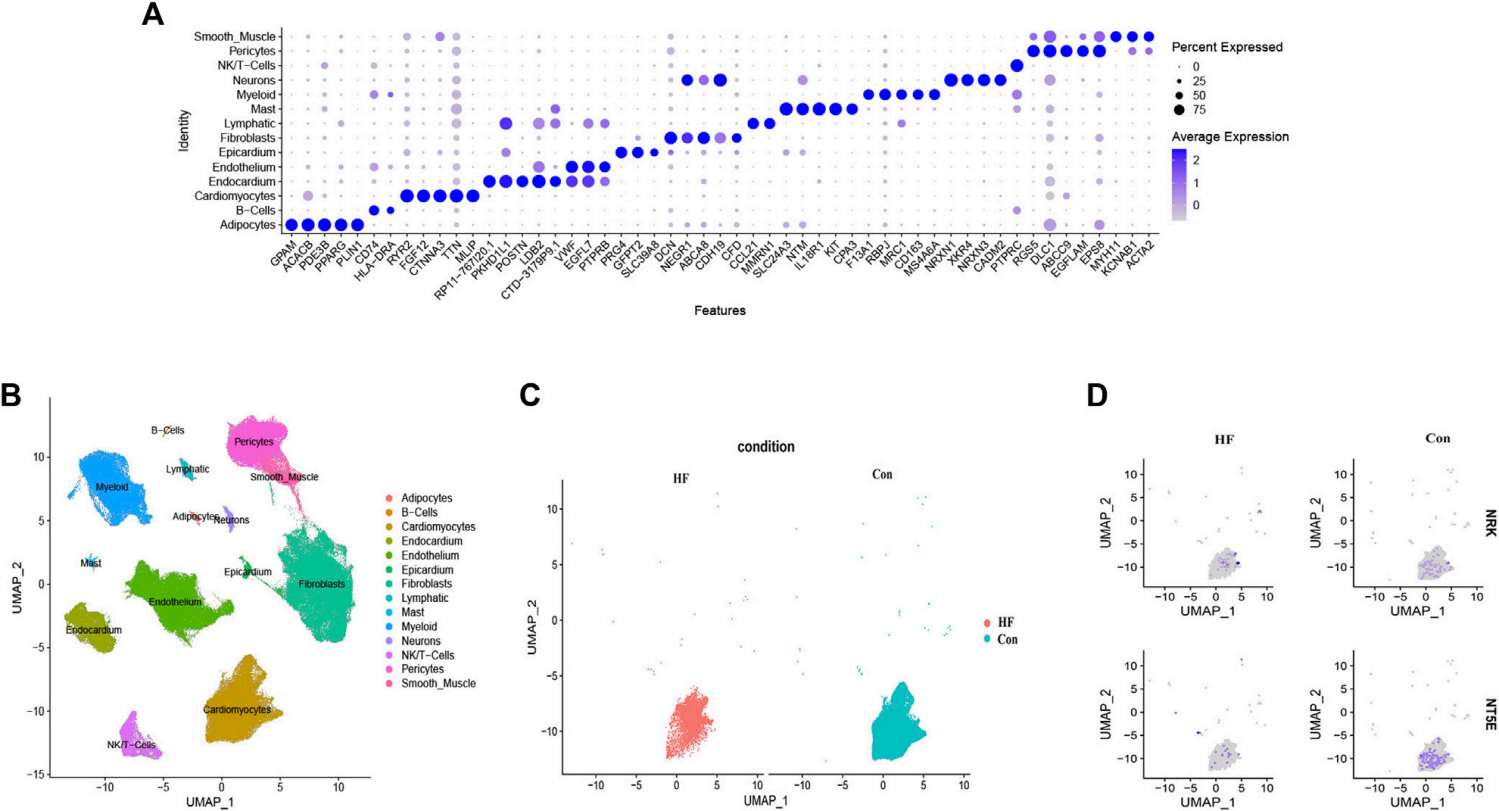
Single-cell RNA-sequencing analysis. **(A)** Clusters annotation and cell types identification via UMAP. **(B)** The expression of the top five marker genes with the most prominence in various types of cells. **(C)** UMAP projection of cardiomyocytes in control and DCM with HF. **(D)** Distribution of NRK and NT5E in cardiomyocytes of control and DCM with HF.

By utilizing cell type marker genes and relevant literature (Shi et al., 2021), we successfully annotated a total of 14 distinct cell types (Figures 7A, B). Notably, we identified and annotated one type of cardiomyocyte in both the control and HF groups (Figure 7C). We then analyzed the expression of NRK and NT5E in cardiomyocytes from both groups. The results revealed that the expression levels of NRK and NT5E were significantly lower in most cardiomyocytes of the HF group compared to those in the control group (Figure 7D). This finding suggests a potential dysregulation of NRK and NT5E expression in cardiomyocytes associated with DCM with HF.

### 3.6 Potential drugs

**FIGURE 8:**
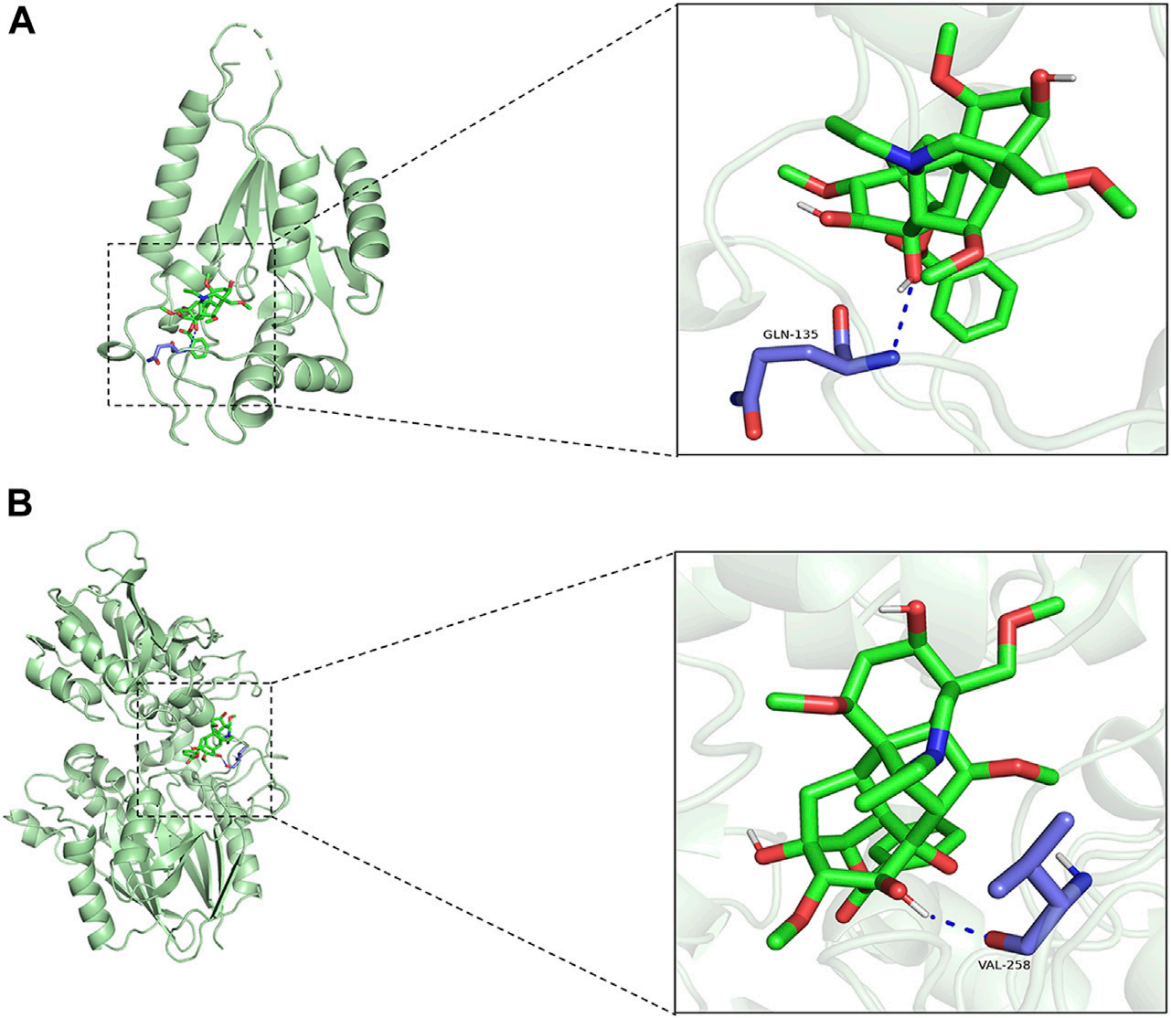
Molecular docking simulations. **(A)** Benzoylaconine interacts with the amino acid residue GLN-135 of NRK. **(B)** Benzoylaconine interacts with the amino acid residue VAL-258 of NT5E.

Aconiti Lateralis Radix Praeparata is a traditional Chinese medicinal herb that has been used extensively in China, especially within the realm of traditional Chinese medicine, for the treatment of various diseases. Aconiti Lateralis Radix Praeparata and its active components are primarily used in the management of rheumatoid arthritis and cardiovascular diseases (Ameri, 1997; Lu et al., 2017; Zhang et al., 2017; Wen et al., 2019a; Wen et al., 2019b; Wen et al., 2020; Xu et al., 2020; Li et al., 2021). Aconiti Lateralis Radix Praeparata contains a diverse array of alkaloids, including aconitine, mesaconitine, hypaconitine, benzoylaconine, benzoylmesaconine, and benzoylhypaconine, among others (Lei et al., 2021). These alkaloids are known for their significant pharmacological effects on the cardiovascular system. In recent years, it has garnered widespread attention in modern pharmacological research, particularly for its potential in treating heart failure. Studies have suggested that aconite could benefit patients with heart failure by improving cardiac function and regulating cardiac metabolism. Although various active components have been identified in aconite, the mechanisms by which it addresses energy metabolism disorders in heart failure are not yet fully elucidated. To identify potential drugs targeting NRK and NT5E, a virtual molecular docking analysis was conducted. We have conducted molecular docking simulations with active components of aconite and the enzymes NRK and NT5E. Benzoylaconine exhibited a binding energy of −7.9 kcal/mol with NRK and −7.5 kcal/mol with NT5E. It interacts with the amino acid residues GLN-135 of NRK, and VAL-258 of NT5E (as shown in Figures 8A, B). Research has confirmed that benzoylaconine can improve mitochondrial function, thereby ameliorating heart failure (Deng et al., 2019; Chen et al., 2022).

Typically, a binding energy less than 0 between a small molecule ligand and its target protein receptor implies spontaneous binding. If the binding energy is lower than −5.0 kcal/mol, it suggests a strong affinity of the small molecule towards the target protein. Given that NRK and NT5E both demonstrate strong affinity for Benzoylaconine, it suggests that Benzoylaconine holds promise as a potential therapeutic agent for HF with DCM.

## 4 Discussion

DCM can lead to varying degrees of HF (Lesizza et al., 2019), placing a significant burden on clinical management. However, the pathogenesis of DCM remains incompletely understood, and effective therapeutic strategies are lacking. In this context, enhancing our understanding of the pathogenic mechanisms underlying dilated cardiomyopathy is of utmost importance, with an urgent need to identify potential therapeutic targets and treatment modalities.

In this study, we employed gene expression profiles from DCM combined with HF patients sourced from the GEO database. Utilizing various bioinformatics methodologies, we screened for genes associated with cardiac energy metabolism in the context of DCM combined with HF. Preliminary analysis was conducted to assess their roles in DCM development and their potential as therapeutic targets. Our research, conducted through GSEA analysis of the original dataset, revealed significant enrichment in ‘metabolic pathways’ and ‘metabolic processes’. Further investigation included DEGs, as well as KEGG and GO analyses, which unveiled enriched pathways such as “taurine and hypotaurine metabolism”, “arachidonic acid metabolism”, and “glycine, serine, and threonine metabolism”, all closely related to myocardial metabolism (West et al., 2016; West et al., 2016). Previous studies have also indicated a connection between DCM and myocardial energy metabolism disturbances. To identify key genes associated with myocardial energy metabolism disturbances in DCM, we extracted energy metabolism-related genes from the database and applied WGCNA to the dataset. This allowed us to pinpoint four highly correlated modules associated with HF. The intersection of these modules’ genes, DEGs, and myocardial energy metabolism genes pointed to two central genes: NRK and NT5E.

NRK and NT5E play a crucial role in NAD^+^ synthesis. Lowered levels of NAD + or changes in the NAD+/NADH redox balance have been noted in HF (Lee et al., 2016; Yoshino et al., 2018; Hu et al., 2020). Research indicates that adding NAD + precursors like NR or NMN could be advantageous for preclinical models or patients with HF (Pillai et al., 2010; Diguet et al., 2018; Zhou et al., 2020; Zapata-Pérez et al., 2021; Wang et al., 2022). However, a recent study evaluating published research on human NR supplements found that oral NR supplementation exhibited minimal associated clinical efficacy (Damgaard and Treebak, 2023). This conclusion does not provide evidence supporting NAD^+^ as a treatment for HF and, to some extent, challenges the value of NAD^+^ in HF therapy. In fact, the premise of supplementing NAD^+^ precursors for HF treatment assumes a deficiency of NAD^+^ in the presence of otherwise normal physiological function. Not all HF patients may only lack NAD^+^ precursors; some may experience issues in the conversion of NAD^+^ precursors to NAD^+^. Therefore, exclusively using NAD^+^ precursors for the treatment of all HF subtypes may be an incomplete consideration.

Transcriptome analysis reveals that NRK and NT5E are upregulated in HF, consistent with existing research (Ren et al., 2018; Byun et al., 2019). However, few researchers have explored the upregulation mechanism of NRK in DCM (Shahzadi et al., 2022). Historically, researchers have not paid much attention to the imbalance of NRK and NT5E in HF, as their positive roles implied that attention should be directed towards downregulation. It is well-known that the conversion of NMN to NAD^+^ relies largely on NRK (Tempel et al., 2007). The loss of NRK expression in tissues may hinder the effectiveness of supplementing NAD^+^ precursors to raise NAD^+^ levels, while NT5E provides NR as the conversion substrate for NRK, underscoring the critical roles of NRK and NT5E in NAD^+^ supplementation therapy for HF. Single-cell sequencing analysis reveals a downregulation of NRK and NT5E levels in cardiomyocytes, in stark contrast to the expression levels in the total heart cells. This observation may genuinely shed light on the true underlying cause of NAD^+^ deficiency in HF. The physiological function of the heart heavily depends on cardiomyocytes. However, previous research has often focused on non-cardiomyocyte cells or the heart as a whole (Zhou et al., 2020; Tong et al., 2021). Due to compensatory mechanisms, NRK and NT5E levels may not decrease throughout the entire heart, and they might even increase in other cell types within the heart, potentially leading researchers to overlook the imbalance of NRK and NT5E. This, to some extent, explains why the therapeutic effects of uniformly supplementing NAD^+^ precursors to HF patients have been questioned. The compensatory upregulation of NRK and NT5E levels in non-cardiomyocyte cells may result in a higher conversion of NAD^+^ precursors to total NAD^+^ after supplementation, which could explain why raising total NAD^+^ levels through precursor supplementation only leads to a limited improvement in HF.

ROC analysis has highlighted the critical roles of NRK and NT5E in the energy metabolism of DCM combined with HF, indicating their potential clinical value in treatment. The decrease in NRK and NT5E levels in cardiomyocytes, accompanied by compensatory upregulation in other heart cell types, suggests that evaluating the diagnostic value of these two genes using heart cells instead of cardiomyocytes is feasible. Subsequently, a comprehensive ceRNA network was constructed using an online search database, pairing lncRNAs, miRNAs, and mRNAs. Three miRNAs targeting NRK mRNA and five miRNAs targeting NT5E mRNA were identified. The top two miRNAs targeting NRK and NT5E were selected to predict their interactions with lncRNAs and circRNAs, revealing that 31 lncRNAs and 50 circRNAs competitively bound to hsa-miR-139-5p to regulate NRK, and 39 lncRNAs and 50 circRNAs competitively bound to hsa-miR-30e-5p to regulate NT5E. This emphasizes the complexity of the regulation of NRK and NT5E in myocardial energy metabolism in HF.

In China, Aconiti Lateralis Radix Praeparata is a commonly used traditional Chinese medicine for treating heart failure (Lu et al., 2017; Wen et al., 2019b; Wen et al., 2020; Xu et al., 2020), but its exact target sites and mechanisms of action have not been fully elucidated. This study, for the first time, conducted docking simulations of Aconiti Lateralis Radix Praeparata’s active components with NRK and NT5E, selecting the complexes with the lowest binding energies. These small molecules may hold potential in treating energy metabolism disorders associated with heart failure and could serve as potential therapeutic drugs.

However, the mechanisms underlying the dysregulation of NRK and NT5E in HF, as well as potential therapeutic drugs, are based on data analysis and virtual molecular docking results. Further *in vivo* and *in vitro* experiments are necessary to validate these findings.

## 5 Conclusion

In this study, we have identified two genes associated with myocardial energy metabolism in DCM with HF and have also identified potential candidate small molecules for treating energy metabolism disorders in HF. The research findings underscore the crucial role of energy metabolism dysfunction in the pathogenesis of DCM with HF. Benzoylaconine holds promise as a therapeutic drug for addressing energy metabolism disorders in DCM with HF by targeting NRK and NT5E. These discoveries provide valuable insights for future research directions and potential therapeutic interventions.

## Data Availability

The datasets presented in this study can be found in online repositories. The names of the repository/repositories and accession number(s) can be found in the article/Supplementary Material.

## Supplementary material

SUPPLEMENTARY FIGURE S1 Normalization process of GEO dataset. **(A)** UMAP plot of the two datasets before normalization. **(B)** UMAP plot of the two datasets after normalization. **(C)** Expression density plot of the two datasets before normalization. **(D)** Expression density plot of the two datasets after normalization. **(E)** Expression distribution plot for the two datasets before normalization. **(F)** Expression distribution plot for the two datasets after normalization.

SUPPLEMENTARY FIGURE S2 Single cell quality control, data cleaning and principal component analysis. **(A)** Volcanic map. **(B)** JackStrawPlot. **(C, D)** FeatureCor.

## References

[B1] Ameri A. (1997). Effects of the alkaloids 6-benzoylheteratisine and heteratisine on neuronal activity in rat hippocampal slices. Neuropharmacology 36 (8), 1039–1046. 10.1016/s0028-3908(97)00095-6 9294968

[B2] Berman H. M. Westbrook J. Feng Z. Gilliland G. Bhat T. N. Weissig H. (2000). The protein data bank. Nucleic Acids Res. 28 (1), 235–242. 10.1093/nar/28.1.235 10592235 PMC102472

[B3] Bieganowski P. Brenner C. (2004). Discoveries of nicotinamide riboside as a nutrient and conserved NRK genes establish a Preiss-Handler independent route to NAD+ in fungi and humans. Cell 117 (4), 495–502. 10.1016/s0092-8674(04)00416-7 15137942

[B4] Byun J. Oka S. I. Imai N. Huang C. Y. Ralda G. Zhai P. (2019). Both gain and loss of Nampt function promote pressure overload-induced heart failure. Am. J. Physiol. Heart Circ. Physiol. 317 (4), H711–H725. 10.1152/ajpheart.00222.2019 31347918 PMC6843022

[B5] Charron P. Elliott P. M. Gimeno J. R. Caforio A. L. P. Kaski J. P. Tavazzi L. (2018). The Cardiomyopathy Registry of the EURObservational Research Programme of the European Society of Cardiology: baseline data and contemporary management of adult patients with cardiomyopathies. Eur. Heart J. 39 (20), 1784–1793. 10.1093/eurheartj/ehx819 29378019

[B6] Chen L. Yan L. Zhang W. (2022). Benzoylaconine improves mitochondrial function in oxygen-glucose deprivation and reperfusion-induced cardiomyocyte injury by activation of the AMPK/PGC-1 axis. Korean J. Physiol. Pharmacol. 26 (5), 325–333. 10.4196/kjpp.2022.26.5.325 36039733 PMC9437369

[B7] Damgaard M. V. Treebak J. T. (2023). What is really known about the effects of nicotinamide riboside supplementation in humans. Sci. Adv. 9 (29), eadi4862. 10.1126/sciadv.adi4862 37478182 PMC10361580

[B8] Deng X. H. Liu J. J. Sun X. J. Dong J. C. Huang J. H. (2019). Benzoylaconine induces mitochondrial biogenesis in mice via activating AMPK signaling cascade. Acta Pharmacol. Sin. 40 (5), 658–665. 10.1038/s41401-018-0174-8 30315253 PMC6786398

[B9] Diguet N. Trammell S. A. J. Tannous C. Deloux R. Piquereau J. Mougenot N. (2018). Nicotinamide riboside preserves cardiac function in a mouse model of dilated cardiomyopathy. Circulation 137 (21), 2256–2273. 10.1161/CIRCULATIONAHA.116.026099 29217642 PMC6954688

[B10] Elliott P. Andersson B. Arbustini E. Bilinska Z. Cecchi F. Charron P. (2008). Classification of the cardiomyopathies: a position statement from the European society of cardiology working group on myocardial and pericardial diseases. Eur. Heart J. 29 (2), 270–276. 10.1093/eurheartj/ehm342 17916581

[B11] He Z. Yang X. Tian X. Li L. Liu M. (2022). Yeast cell surface engineering of a nicotinamide riboside kinase for the production of β-nicotinamide mononucleotide via whole-cell catalysis. ACS Synth. Biol. 11 (10), 3451–3459. 10.1021/acssynbio.2c00350 36219824

[B12] Hu Q. Zhang H. Gutiérrez Cortés N. Wu D. Wang P. Zhang J. (2020). Increased drp1 acetylation by lipid overload induces cardiomyocyte death and heart dysfunction. Circ. Res. 126 (4), 456–470. 10.1161/CIRCRESAHA.119.315252 31896304 PMC7035202

[B13] Khomtchouk B. B. Tran D. T. Vand K. A. Might M. Gozani O. Assimes T. L. (2020). Cardioinformatics: the nexus of bioinformatics and precision cardiology. Brief. Bioinform 21 (6), 2031–2051. 10.1093/bib/bbz119 31802103 PMC7947182

[B14] Langfelder P. Horvath S. (2008). WGCNA: an R package for weighted correlation network analysis. BMC Bioinforma. 9, 559. 10.1186/1471-2105-9-559 PMC2631488 19114008

[B15] Larocca T. J. Seeger T. Prado M. Perea-Gil I. Karakikes I. Mecham B. H. (2020). Pharmacological silencing of microRNA-152 prevents pressure overload–induced heart failure. Circ. Heart Fail 13 (3), e006298. 10.1161/CIRCHEARTFAILURE.119.006298 32160771 PMC7439562

[B16] Lee C. F. Chavez J. D. Garcia-Menendez L. Choi Y. Roe N. D. Chiao Y. A. (2016). Normalization of NAD+ redox balance as a therapy for heart failure. Circulation 134 (12), 883–894. 10.1161/CIRCULATIONAHA.116.022495 27489254 PMC5193133

[B17] Lei H. Zhang Y. Ye J. Cheng T. Liang Y. Zu X. (2021). A comprehensive quality evaluation of Fuzi and its processed product through integration of UPLC-QTOF/MS combined MS/MS-based mass spectral molecular networking with multivariate statistical analysis and HPLC-MS/MS. J. Ethnopharmacol. 266, 113455. 10.1016/j.jep.2020.113455 33039630

[B18] Lesizza P. Aleksova A. Ortis B. Beltrami A. P. Giacca M. Sinagra G. (2019). “Regenerative medicine and biomarkers for dilated cardiomyopathy,” in Dilated cardiomyopathy: from genetics to clinical management. Editors Sinagra G. Merlo M. Pinamonti B. (Switzerland: Cham: Springer Cham), 173–185. 32091713

[B19] Li S. Li R. Xu Y. X. Baak J. P. A. Gao J. H. Shu J. Q. (2021). Traditional Chinese medicine aconiti radix cocta improves rheumatoid arthritis via suppressing COX-1 and COX-2. Evid. Based Complement. Altern. Med. 2021, 5523870. 10.1155/2021/5523870 PMC8443343 34539799

[B20] Lu X. Zhang L. Li P. Wang J. Li R. Huang Y. (2017). The protective effects of compatibility of Aconiti Lateralis Radix Praeparata and Zingiberis Rhizoma on rats with heart failure by enhancing mitochondrial biogenesis via Sirt1/PGC-1α pathway. Biomed. Pharmacother. 92, 651–660. 10.1016/j.biopha.2017.05.117 28578259

[B21] Luczak E. D. Wu Y. Granger J. M. Joiner M. A. Wilson N. R. Gupta A. (2020). Mitochondrial CaMKII causes adverse metabolic reprogramming and dilated cardiomyopathy. Nat. Commun. 11 (1), 4416. 10.1038/s41467-020-18165-6 32887881 PMC7473864

[B22] McKenna W. J. Maron B. J. Thiene G. (2017). Classification, epidemiology, and global burden of cardiomyopathies. Circ. Res. 121 (7), 722–730. 10.1161/CIRCRESAHA.117.309711 28912179

[B23] Minor M. Alcedo K. P. Battaglia R. A. Snider N. T. (2019). Cell type- and tissue-specific functions of ecto-5'-nucleotidase (CD73). Am. J. Physiol. Cell Physiol. 317 (6), C1079–C1092. 10.1152/ajpcell.00285.2019 31461341 PMC6957383

[B24] Morris G. M. Huey R. Lindstrom W. Sanner M. F. Belew R. K. Goodsell D. S. (2009). AutoDock4 and AutoDockTools4: automated docking with selective receptor flexibility. J. Comput. Chem. 30 (16), 2785–2791. 10.1002/jcc.21256 19399780 PMC2760638

[B25] Pillai V. B. Sundaresan N. R. Kim G. Gupta M. Rajamohan S. B. Pillai J. B. (2010). Exogenous NAD blocks cardiac hypertrophic response via activation of the SIRT3-LKB1-AMP-activated kinase pathway. J. Biol. Chem. 285 (5), 3133–3144. 10.1074/jbc.M109.077271 19940131 PMC2823454

[B26] Ren W. Gao S. Zhang H. Ren Y. Yu X. Lin W. (2018). Decomposing the mechanism of qishen granules in the treatment of heart failure by a quantitative pathway analysis method. Molecules 23 (7), 1829. 10.3390/molecules23071829 30041436 PMC6100320

[B27] Robin X. Turck N. Hainard A. Tiberti N. Lisacek F. Sanchez J. C. (2011). pROC: an open-source package for R and S+ to analyze and compare ROC curves. BMC Bioinforma. 12, 77. 10.1186/1471-2105-12-77 PMC3068975 21414208

[B28] Seferović P. M. Polovina M. Bauersachs J. Arad M. Ben Gal T. Lund L. H. (2019). Heart failure in cardiomyopathies: a position paper from the heart failure association of the European society of cardiology. Eur. J. Heart Fail 21 (5), 553–576. 10.1002/ejhf.1461 30989768

[B29] Shahzadi S. K. Marzook H. Qaisar R. Ahmad F. (2022). Nicotinamide riboside kinase-2 inhibits JNK pathway and limits dilated cardiomyopathy in mice with chronic pressure overload. Clin. Sci. (Lond). 136 (2), 181–196. 10.1042/CS20210964 35048952

[B30] Sharma S. Bhattarai S. Ara H. Sun G. St Clair D. K. Bhuiyan M. S. (2020). SOD2 deficiency in cardiomyocytes defines defective mitochondrial bioenergetics as a cause of lethal dilated cardiomyopathy. Redox Biol. 37, 101740. 10.1016/j.redox.2020.101740 33049519 PMC7559509

[B31] Shi X. Zhang L. Li Y. Xue J. Liang F. Ni H. W. (2021). Integrative analysis of bulk and single-cell RNA sequencing data reveals cell types involved in heart failure. Front. Bioeng. Biotechnol. 21 (5), 553–576. 10.1002/ejhf.1461 PMC8766768 35071201

[B32] Sociali G. Raffaghello L. Magnone M. Zamporlini F. Emionite L. Sturla L. (2016). Antitumor effect of combined NAMPT and CD73 inhibition in an ovarian cancer model. Oncotarget 7 (3), 2968–2984. 10.18632/oncotarget.6502 26658104 PMC4823084

[B33] Tempel W. Rabeh W. M. Bogan K. L. Belenky P. Wojcik M. Seidle H. F. (2007). Nicotinamide riboside kinase structures reveal new pathways to NAD+. PLoS Biol. 5 (10), e263. 10.1371/journal.pbio.0050263 17914902 PMC1994991

[B34] Tong D. Schiattarella G. G. Jiang N. Altamirano F. Szweda P. A. Elnwasany A. (2021). NAD(+) repletion reverses heart failure with preserved ejection fraction. Circ. Res. 128 (11), 1629–1641. 10.1161/CIRCRESAHA.120.317046 33882692 PMC8159891

[B35] Trott O. Olson A. (2010). AutoDock Vina: improving the speed and accuracy of docking with a new scoring function, efficient optimization, and multithreading. J. Comput. Chem. 31 (2), 455–461. 10.1002/jcc.21334 19499576 PMC3041641

[B36] Vignoli A. Fornaro A. Tenori L. Castelli G. Cecconi E. Olivotto I. (2022). Metabolomics fingerprint predicts risk of death in dilated cardiomyopathy and heart failure. Front. Cardiovasc Med. 9, 851905. 10.3389/fcvm.2022.851905 35463749 PMC9021397

[B37] Wang D. D. Airhart S. E. Zhou B. Shireman L. M. Jiang S. Melendez Rodriguez C. (2022). Safety and tolerability of nicotinamide riboside in heart failure with reduced ejection fraction. JACC Basic Transl. Sci. 7 (12), 1183–1196. 10.1016/j.jacbts.2022.06.012 36644285 PMC9831861

[B38] Wen J. Zhang L. Liu H. Wang J. Li J. Yang Y. (2019a). Salsolinol attenuates doxorubicin-induced chronic heart failure in rats and improves mitochondrial function in H9c2 cardiomyocytes. Front. Pharmacol. 10, 1135. 10.3389/fphar.2019.01135 31680945 PMC6797600

[B39] Wen J. Zhang L. Wang J. Wang J. Wang L. Wang R. (2020). Therapeutic effects of higenamine combined with [6]-gingerol on chronic heart failure induced by doxorubicin via ameliorating mitochondrial function. J. Cell Mol. Med. 24 (7), 4036–4050. 10.1111/jcmm.15041 32073745 PMC7171398

[B40] Wen J. Zou W. Wang R. Liu H. Yang Y. Li H. (2019b). Cardioprotective effects of Aconiti Lateralis Radix Praeparata combined with Zingiberis Rhizoma on doxorubicin-induced chronic heart failure in rats and potential mechanisms. J. Ethnopharmacol. 238, 111880. 10.1016/j.jep.2019.111880 31004728

[B41] West J. A. Beqqali A. Ament Z. Elliott P. Pinto Y. M. Arbustini E. (2016). A targeted metabolomics assay for cardiac metabolism and demonstration using a mouse model of dilated cardiomyopathy. Metabolomics 12, 59. 10.1007/s11306-016-0956-2 27069442 PMC4781888

[B42] Xu X. Xie X. Zhang H. Wang P. Li G. Chen J. (2020). Water-soluble alkaloids extracted from Aconiti Radix lateralis praeparata protect against chronic heart failure in rats via a calcium signaling pathway. Biomed. Pharmacother. 135, 111184. 10.1016/j.biopha.2020.111184 33418305

[B43] Yoshino J. Baur J. A. Imai S. I. (2018). NAD(+) Intermediates: the biology and therapeutic potential of NMN and NR. Cell Metab. 27 (3), 513–528. 10.1016/j.cmet.2017.11.002 29249689 PMC5842119

[B44] Yu G. Wang L. G. Han Y. He Q. Y. (2012). ClusterProfiler: an R package for comparing biological themes among gene clusters. OMICS 16 (5), 284–287. 10.1089/omi.2011.0118 22455463 PMC3339379

[B45] Yu Y. D. Xue Y. T. Li Y. (2023). Identification and verification of feature biomarkers associated in heart failure by bioinformatics analysis. Sci. Rep. 13 (1), 3488. 10.1038/s41598-023-30666-0 36859608 PMC9977868

[B46] Zapata-Pérez R. Tammaro A. Schomakers B. V. Scantlebery A. M. L. Denis S. Elfrink H. L. (2021). Reduced nicotinamide mononucleotide is a new and potent NAD(+) precursor in mammalian cells and mice. FASEB J. 35 (4), e21456. 10.1096/fj.202001826R 33724555

[B47] Zhang L. Lu X. Wang J. Li P. Li H. Wei S. (2017). Zingiberis rhizoma mediated enhancement of the pharmacological effect of aconiti lateralis radix praeparata against acute heart failure and the underlying biological mechanisms. Biomed. Pharmacother. 96, 246–255. 10.1016/j.biopha.2017.09.145 28987949

[B48] Zhou B. Wang D. D. Qiu Y. Airhart S. Liu Y. Stempien-Otero A. (2020). Boosting NAD level suppresses inflammatory activation of PBMCs in heart failure. J. Clin. Invest. 130 (11), 6054–6063. 10.1172/JCI138538 32790648 PMC7598081

[B49] Zhou Q. Yu B. Anderson C. Huang Z. P. Hanus J. Zhang W. (2019). LncEGFL7OS regulates human angiogenesis by interacting with MAX at the EGFL7/miR-126 locus. eLife 82, e40470. 10.7554/eLife.40470 PMC6370342 30741632

