# Supplementary material for: Bioinformatics identifies key genes and potential drugs for energy metabolism disorders in heart failure with dilated cardiomyopathy
Source: Front Pharmacol. 2024 Mar 6;15:1367848. doi: 10.3389/fphar.2024.1367848 (PMC10952830; doi:10.3389/fphar.2024.1367848)
Supplement: Supplementary file 1 [file Table1.DOC]

All data have been uploaded. Please click the link below:

https://www.jianguoyun.com/p/DQa-c8sQvPKYDBjBg6kFIAA
